# WNT Signaling in Tumors: The Way to Evade Drugs and Immunity

**DOI:** 10.3389/fimmu.2019.02854

**Published:** 2019-12-20

**Authors:** Elena Martin-Orozco, Ana Sanchez-Fernandez, Irene Ortiz-Parra, Maria Ayala-San Nicolas

**Affiliations:** ^1^Department of Biochemistry and Molecular Biology (B) and Immunology, School of Medicine, University of Murcia, Murcia, Spain; ^2^Biomedical Research Institute of Murcia (IMIB), ARADyAL, Murcia, Spain

**Keywords:** WNT, β-catenin, ABCB1, PD-L1, CD47, multidrug resistance (MDR), immunity escape, cancer

## Abstract

WNT/β-catenin signaling is involved in many physiological processes. Its implication in embryonic development, cell migration, and polarization has been shown. Nevertheless, alterations in this signaling have also been related with pathological events such as sustaining and proliferating the cancer stem cell (CSC) subset present in the tumor bulk. Related with this, WNT signaling has been associated with the maintenance, expansion, and epithelial-mesenchymal transition of stem cells, and furthermore with two distinctive features of this tumor population: therapeutic resistance (MDR, multidrug resistance) and immune escape. These mechanisms are developed and maintained by WNT activation through the transcriptional control of the genes involved in such processes. This review focuses on the description of the best known WNT pathways and the molecules involved in them. Special attention is given to the WNT cascade proteins deregulated in tumors, which have a decisive role in tumor survival. Some of these proteins function as extrusion pumps that, in the course of chemotherapy, expel the drugs from the cells; others help the tumoral cells hide from the immune effector mechanisms. Among the WNT targets involved in drug resistance, the drug extrusion pump MDR-1 (P-GP, ABCB1) and the cell adhesion molecules from the CD44 family are highlighted. The chemokine CCL4 and the immune checkpoint proteins CD47 and PD-L1 are included in the list of WNT target molecules with a role in immunity escape. This pathway should be a main target in cancer therapy as WNT signaling activation is essential for tumor progression and survival, even in the presence of the anti-tumoral immune response and/or antineoplastic drugs. The appropriate design and combination of anti-tumoral strategies, based on the modulation of WNT mediators and/or protein targets, could negatively affect the growth of tumoral cells, improving the efficacy of these types of therapies.

## Introduction

Tumors consist of a heterogeneous mix of cellular populations composed of a small number of cancer stem cells, stromal cells, and tumor infiltrating immune cells, among others. Supporting this idea, several observations have indicated that each specific tumor contains subclones with a wide assortment of gene mutations and promoter hypermethylation in all the cells that constitute the tumor and its microenvironment (1–3).

Currently, two proposals seek to explain tumor heterogeneity. The clonal evolution model proposes that arbitrary mutations in each tumor could generate clones with acquired advantages under adverse selection requirements such as oxygen and/or nutrients. Such clones will expand while the others, less adapted to these conditions, will disappear. Different requirements for tumor growth may be present in different areas of the tumor or at different times or, furthermore, clones with better resistance mechanisms could became advantageous after therapy application. With this proposal, all the cells within a tumor could regenerate the tumor in a different location. The cancer stem cell (CSC) model assumes that only the subset of cancer stem cells on each tumor possesses the capacity to initiate and maintain tumor growth (4). Thus, CSCs are responsible for tumor heterogeneity by generating a variety of cell types that can be reverted, since the terminally differentiated cells can go back and gain CSCs properties under specific conditions. This concept of cellular plasticity unifies the two current models proposed (5–10).

Normal stem or differentiated cells could originate CSCs through a process of consecutive mutations that lead to the acquisition of characteristic cancer stem cell properties of self-renewal, pluripotency, tumor reconstitution capacity, chemo-resistance, low immunogenicity, and/or immune escape capacity. Drug resistance could be mediated by several mechanisms such as the acquisition of quiescence, improved DNA repair, drug efflux capacity, decreased apoptosis, and interaction with the microenvironment (11). Low immunogenicity and/or immunity evasion could be acquired by different strategies such as the production of immunosuppressive molecules; the recruitment of regulatory cells of the immune system; the expression of inhibitory molecules of the anti-tumoral immune response; the loss of tumor antigen expression, the downregulation of major histocompatibility complex I and II (MHC-I and MHC-II) expression, and/or the inhibition of co-stimulatory molecules on antigen presenting cells. These characteristics, mean that the CSCs are the main players in tumor initiation, progression, invasion, metastasis, drug-resistance, and recurrence (11).

The oncogenic properties of CSCs require a number of developmental pathways previously associated to the regulation of normal stem cells (12). Among them, the WNT pathway is deregulated in epithelial-mesenchymal transition (EMT) and CSCs (13). Mutations in WNT signaling components, such as APC, AXIN, β-catenin, and WNT ligands were first observed in colorectal cancers, but have also been reported in other solid tumors (14–21) and hematological malignancies, including leukemia and Multiple Myeloma (MM) (22–24).

There are different WNT pathways interconnected with each other which have been classified as canonical (β-catenin-dependent) and non-canonical signaling pathways. In general, it is assumed that the canonical WNT cascade is responsible for self-renewal, proliferation, or differentiation of progenitor cells, and that the non-canonical cascade participates in the maintenance of stem cells, cell movement, or inhibition of the canonical pathway (25).

## WNT Signaling Components and Pathways

### WNT Ligands, Receptors, and Co-receptors

The WNT ligands family is constituted by 19 secreted glycoproteins that can participate in one or several WNT signaling pathways in a manner that can be autocrine or paracrine. They exhibit specific expression patterns and functions and are highly conserved from invertebrates to mammals (26, 27). All WNT ligands become glycosylated in the endoplasmic reticulum (ER); and also acylated by the O-acetyltransferase Porcupine (PRCN) (28–31). Next, lipid-modified WNT ligands bind to the transmembrane protein Evenness interrupted WNTless (Evi/WIs) and are transported to the plasma membrane *via* the Golgi apparatus with the assistance of the p24 proteins (32–34). Finally, the transportation of WNT ligands on the extracellular space occurs in membrane enclosed vesicles such as exosomes (28, 31, 35).

The family of Frizzled (FZD) receptors interacts with WNT ligands and with the co-receptor's low-density lipoprotein receptor-related proteins 5,6 (LRP5/6). While the complex consisting of WNT, FZD, and LRP proteins activates the canonical WNT/β-catenin signaling cascade, the complex formed by FZD and/or ROR1/ROR2/RYK (Receptor tyrosine kinase-like orphan receptor) receptors activates non-canonical WNT signaling cascades (WNT/PCP or planar cell polarity and the WNT/Ca2+ signaling cascades). The complex WNT-FZD-LRP also activates the WNT/STOP (stabilization of proteins) route which is a subtype of the non-canonical WNT signaling pathway which decelerates protein degradation when cells prepare to divide during mitosis (36–38).

### WNT Canonical Pathway: On and Off

**Figure 1:**
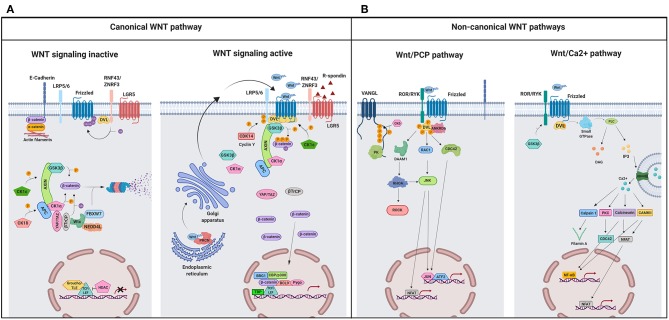
A schematic illustration representing different WNT signaling pathways. **(A)** Canonical WNT signaling. Left panel shows inactive pathway. In the absence of WNT ligands, β-catenin is phosphorylated by the destruction complex, constituted by the scaffolding proteins APC and AXIN and the kinases GSK3β and CK1α. Then, β-catenin is ubiquitinated and targeted for proteasomal degradation by the complex containing β-TrCP, FBXW7, NEDDL4, and WTX proteins. Thus, β-catenin degradation prevents its presence in the nucleus where a complex formed by TCF/LEF and TLE/Groucho binds HDACs to inhibit transcription of target genes. Right panel shows canonical WNT signaling active. The binding of WNT ligands to FZD receptors and LRP co-receptors activates WNT signaling. LRP receptors are phosphorylated by CK1α and GSK3β. Then, DVL proteins polymerize and are activated at the plasma membrane inhibiting the destruction complex. This results in stabilization and accumulation of β-catenin in the cytosol and its subsequent translocation into the nucleus where it displaces TLE/Groucho repressors forming an active complex with TCF/LEF proteins that bind co-activators such as CBP/p300, BRG1, BCL9, and PYGO. An alternative way of β-catenin signaling includes the disruption of epithelial E-cadherin interactions, which breaks the binding of β-catenin to the cytoplasmic domain of cadherin and leads to the accumulation of β-catenin first in the cytosol, and later in the nucleus. **(B)** Schematic illustration representing the main non-canonical WNT pathways. Left panel shows the WNT/PCP pathway. WNT ligands bind to the FZD receptor and the co-receptors ROR 1/2 (or RYK). Then, DVL is recruited and activated followed by VANGL activation. Then DVL binds to the small GTPase RHO A with the collaboration of the cytoplasmic protein DAAM1. The small GTPases RAC1 and RHO activate ROCK and JNK. This leads to rearrangements of the cytoskeleton and/or transcriptional responses via for example, ATF2 and/or NFAT. Right panel shows the WNT/Ca2+ pathway. The signaling is initiated when WNT ligands bind to the FZD receptor and the co-receptor ROR 1/2 (or RYK). Then, DVL is recruited and activated and binds to the small GTPase which activates phospholipase C leading to intracellular calcium fluxes and downstream calcium dependent cytoskeletal and/or transcriptional responses. APC, adenomatous polyposis coli; BCL9, B-cell CLL/lymphoma 9 protein; β-TrCP, β-Transducin repeat-containing protein; BRG1, Brahma related gene 1; CAMKII, calmodulin-dependent protein kinase II; CBP, CREB-binding protein; CDC42, cell division control protein 42; CELSR, cadherin EGF LAG seven-pass G-type receptor; CK1α,ε,δ, casein kinase 1α,ε,δ; DAAM1, DVL associated activator of morphogenesis; DAG, diacylglycerol; DVL, disheveled; FBXW7, F box/WD repeat-containing protein 7; FZD, Frizzled; GSK3β, glycogen synthase kinase 3β; IP3, inositol 1,4,5 triphosphate; JNK, JUN kinase; LGR5, Leucine-rich repeat-containing G-protein-coupled receptor 5; LRP5/6, low-density lipoprotein receptor-related protein 5/6; NEDD4L, neural precursor cell expressed, developmentally downregulated 4-like; NFAT, nuclear factor of activated T cells; NF-κB, nuclear factor kappa B; PK, Prickle; PKC, protein kinase C; PLC, Phospholipase C; p300, E1A Binding Protein p300; RAC, Ras-related C3 botulinum toxin substrate; RHOA, Ras homolog gene family member A; ROCK, Rho kinase; ROR1/2, bind tyrosine kinase-like orphan receptor 1 or 2; RYK, receptor-like tyrosine kinase; TBP, TATA-binding protein; PRCN, Porcupine; PYGO, Pygopus; RNF43, Ring finger protein 43; RSPO, R-spondin; TCF/LEF, T-cell factor/lymphoid enhancer factor; TLE, Transducin-Like Enhancer of Split proteins; VANGL, Van Gogh-like; WTX, Wilms tumor suppressor protein complex; YAP/TAZ, Yes-associated protein/Transcriptional co-activator with a PDZ-binding domain; ZNRF3, Zinc and Ring Finger 3. Created with BioRender.com.

The central point of this pathway is the activation of the protein β-catenin, which can be found in the cell in different forms and locations. Thus, at the cytoplasmic membrane, β-catenin remains associated with E-cadherin and, through α-catenin, connects actin filaments to form the cytoskeleton (Figure 1A, left panel); in the cytoplasm, β-catenin levels are strictly controlled; and in the nucleus this protein regulates transcriptional activation and chromatin remodeling.

In the absence of ligands, the WNT pathway is inactive (Figure 1A, left panel) and β-catenin is continuously synthesized, ubiquitinated, and degraded in the cytosol by a destruction complex constituted by the two scaffold proteins, adenomatous polyposis coli (APC) and axis inhibition protein 1 (AXIN1), Ser/Thr kinases such as casein kinase 1 (CK1α, ε y δ) and glycogen synthase kinase 3β (GSK3β), and two transcriptional regulators (YAP/TAZ; Yes-associated protein/transcriptional co-activator with a PDZ-binding domain) of the Hippo pathway (37–39). Thus, β-catenin will not be available for nuclear transport and transcriptional regulation.

On the contrary, when WNT binds to the FZD receptor, the canonical WNT pathway becomes active (Figure 1A, right panel) and the recruitment of co-receptors LRP5/6 changes AXIN conformation, impairing its interaction with β-catenin and preventing the phosphorylation of this protein (40, 41). Alternatively, Li et al. (42) propose a model in which the destruction complex can bind and phosphorylate β-catenin, but the absence of β-TrCP within the complex prevents ubiquitination and degradation of the protein (38, 43), so that β-catenin accumulates in the cytosol and travels to the nucleus, where it induces the transcription of specific genes (Figure 1A, right panel) (40, 43–46) (listed in http://www.stanford.edu/Brnusse/WNTwindow.html).

### Non-canonical or Alternative WNT Signaling Pathways

Non-canonical WNT pathways do not require β-catenin stabilization and the signal initiates through the binding of WNT to the FZD receptor without LRP co-receptor participation. Although several non-canonical WNT pathways have been identified, the best known are the WNT/PCP and the WNT/Ca2+ pathways (Figure 1B).

WNT/PCP signaling is implicated in the establishment of cell polarity and cell migration (31). In this pathway (Figure 1B, left panel), the binding of the WNT ligand initiates a cascade in which ROR/RYK helps to transmit the signal to VANGL2 and induces its phosphorylation (47–50). Additionally, RHOA and RAC activate JUN kinase (JNK), which can induce gene transcription through the activation of AP1 (jun-ATF-2) and the nuclear factor of activated T cells (NFAT) (Figure 1B, left panel). A crosstalk exists between WNT/PCP and the canonical WNT pathway as it has been shown that VANG-1/VANGL negatively regulates the WNT/β-catenin signaling by a mechanism dependent on DVL (51).

The WNT/Ca2+ signaling pathway (Figure 1B, right panel) is activated by the binding of the WNT ligands to the FZD family of proteins, which in turn activates phospholipase C (PLC). The secondary messengers induce the release of intracellular calcium and then calcium dependent kinases such as calpain-1 and calcineurin (Cn) are activated. These kinases activate the expression of transcription factors such as NFAT and nuclear factor kappa B (NFκB). This pathway is fundamental in the regulation of cell adhesion, cell migration, and embryonic development (25).

### Modulators of WNT Signaling: RNF43/ZNRF3, RSPO, YAP/TAZ, and VANGL

RNF43 (Ring finger protein 43) and its homolog ZNRF3 (Zinc and Ring Finger 3) promote poly-ubiquitination of lysines in the cytoplasmic sequence of FZDs proteins (52, 53) inducing endocytosis and the destruction of these receptors at the lysosome (Figure 1A) (28).

Another WNT regulator is Leucine-rich repeat-containing G-protein-coupled receptor 5 (LGR5), a protein that binds to the R-spondin ligand family members (RSPO) and maintains WNT signaling through the neutralization of the RNF43/ZNF3 ligases (Figure 1A) (36, 37, 54, 55). YAP/TAZ are also WNT regulators and compete with LRP5/6 for the same binding domain of AXIN. Thus, the association of AXIN to YAP/TAZ is incompatible with its association to LRP5/6 (Figure 1A, right panel) (56). Finally, Van Gogh-like (VANGL) and Prickle (PK) are two crucial components of the planar cell polarity pathway and antagonists to the canonical WNT/β-catenin pathway (Figure 1B, left panel). Therefore, the absence of VANGL1/2 decreases the response threshold to WNT3a, without initiating activation of canonical WNT signaling however (51).

## WNT Signaling Activation in Healthy Immune Response and in Tumor Cell Survival

**Figure 2:**
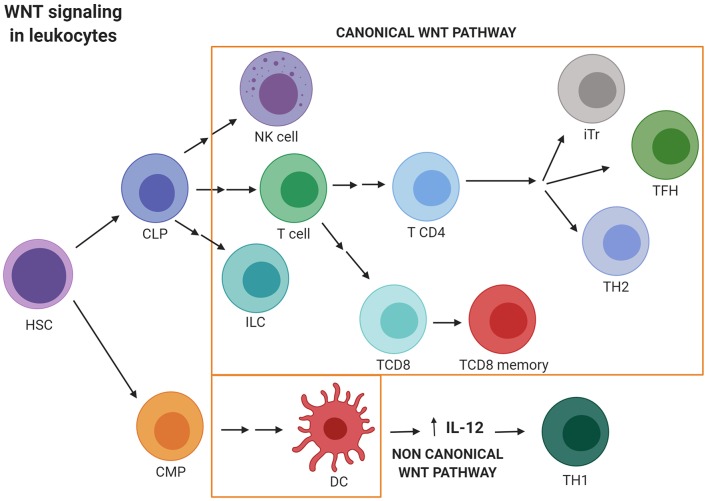
WNT signaling activation in leukocytes. Canonical WNT signaling promotes T cell lymphopoiesis and regulates peripheral T cell activation and differentiation. Thus, canonical WNT activity promotes differentiation into the TH2 and TFH subsets. Regulatory T (Tr) cells survival is also promoted by the canonical WNT pathway. Nevertheless, TCF1 limits the suppressive activity exerted by Tr in the effector T cell population. Finally, WNT/β-catenin activation is essential for the development of innate lymphoid cells (ILCs) natural killer (NK) cells and DC differentiation. Conversely, non-canonical WNT signaling induces the secretion of IL-12 by dendritic cells favoring TH1 responses. CLP, Common lymphoid progenitor; CMP, Common myeloid progenitor; DC, Dendritic cell; ILC, Innate lymphoid cell; iTr, induced regulatory T cell; HSC, hematopoietic stem cell; NK, Natural killer; TFH, T follicular helper cell; TH, T helper. Created with BioRender.com.

WNT signaling should be considered a multifactorial pathway which regulates the transcription of genes that participate in most of the mechanisms used by healthy cells to differentiate and survive. Supporting this, WNT signaling plays a main role in cellular homeostasis, regulating immune cell development and function. Thus, the non-canonical WNT pathway inhibits the differentiation of quiescent HSCs by controlling β-catenin activation (57, 58). Conversely, canonical WNT signaling promotes T cell lymphopoiesis (5, 59–61). The role of WNT signaling in immunity also includes the regulation of peripheral T cell activation and differentiation. As evidence of this, TCF activity has been shown to play crucial roles in the differentiation of memory CD8+ T cells as well as in inducing specific TH subset responses (59, 61). In this sense, the non-canonical WNT signaling by WNT5A induces the secretion of IL-12, by dendritic cells favoring TH1 responses (59, 61–63). However, activation of the canonical pathway promotes TH2 polarization. Specifically, TCF1 inhibits differentiation of naive CD4+ T cells into TH1 and TH17 cells and promotes differentiation into the TH2 and TFH (T follicular helper cells) subsets. Regulatory T (Tr) cell survival is also promoted by the canonical WNT pathway, whilst the effector function and development of TH17 cells are inhibited. Nevertheless, TCF1 also limits the suppressive activity exerted by regulatory T cells in the effector T cell population. Finally, TCF1 is essential for the development of innate lymphoid cells (ILCs) natural killer (NK) cells and DC differentiation (Figure 2) (59, 61, 64, 65).

Since WNT signaling is crucial to immune homeostasis, alterations of this pathway in pathologies such as cancer can also be associated with the profound dysregulation of protective anti-tumoral immune responses [reviewed in (45, 66, 67)]. In fact, WNT signaling is associated with the onset of many types of solid and liquid tumors. As an example, mutations involved in the WNT signaling pathway were found in more than 90% of carcinomas of the colon and rectum [reviewed in (68)]. Related to that, it is broadly accepted that the first step in the progression to CRC frequently consists of APC gene mutations associated with the activation of the canonical WNT pathway. Supporting this, adenoma regression was observed in a murine model when APC function was recovered. Additionally, activating mutations in β-catenin, mediated or not by APC mutations, were observed in 80% of cases of CRC (69). RNF43 mutations causing WNT signaling activation were found in over 18% of colorectal and endometrial cancers (70, 71) which were extremely dependent on the WNT pathway. Since RNF43 mutations make the PDAC cell lines sensitive to treatment with Porcupine inhibitors, the growth of these tumors also relies on WNT ligand secretion. Conversely, induction of DKK1, a WNT antagonist, or treatment with an FZD-specific antibody delays PDAC development (72). Additionally, RSPO translocations are described in 4–18% of patients with gastric, ovarian, and endometrial cancer and about 9% of colorectal cancers (70). Recently, ROR1 and ROR2 co-receptor upregulation was associated to the two different melanoma states: proliferative and invasive. These phenotypes are in part the result of the balance between canonical and non-canonical WNT activation. The treatment of proliferative melanoma cells, expressing ROR1, with WNT5a induced ROR1 degradation, increased ROR2 expression and high invasiveness *in vivo*. (65). WNT signaling activation is also observed in ~50% of patients with breast cancer and is associated with a decrease in overall survival. Although only a small percentage of tumors carry mutations of the main WNT pathway regulators, canonical WNT ligands and receptors are frequently overexpressed, whereas antagonists are downregulated. Among all the WNT regulators, high LGR4 expression was correlated with low patient survival. In Lgr4-deficient mouse models, researchers found a significant delay in mammary tumor development, proliferation, and the occurrence of metastasis. Further molecular analysis demonstrated that LGR4 knockdown inhibited WNT/β-catenin signaling, and the expression of epithelial–mesenchymal transition (EMT) mediators. Finally, there was a 90% decline in the number of CSCs in human breast-cancer cells and in the mouse mammary tumor virus (MMTV)-WNT1 transgenic mouse model. Thus, WNT signaling is implicated in the maintenance of mammary stem cells and breast-cancer stem cells (73–76). Supporting these results, in non-small cell lung cancer it has been shown that maintaining the lung cancer stem cell population requires the degradation of the WNT negative regulators, increasing β-catenin mediated WNT activity (77). Furthermore, WNT signaling plays a critical role in the maintenance and propagation of ovarian cancer stem cells (78); it is critical in desmoid tumor formation (79), and in head and neck squamous carcinomas (HNSCC), where researchers have shown CSC elimination after WNT/β-catenin signaling was downregulated.

WNT signaling deregulation also plays an important role in the development of hematological tumors. WNT ligands and receptors are expressed in the hematopoietic stem cells (HSC) and are present in the bone marrow (59). Thus, the WNT/β-catenin pathway is highly activated in AML (80–82), CML (83, 84), Multiple myeloma (MM), and other types of leukemia. In fact, in mouse models of AML, the WNT/β-catenin signaling pathway is required for leukemia stem cells (LSCs) self-renewal (85). Similarly, a high expression of β-catenin and WNT pathway associated genes has been observed in LSCs in CML (86). Deregulation of the WNT pathway is also associated with abnormal expression of LGR4 in cells isolated from MM patients (87, 88).

WNT signaling has been involved not only in tumor development but also in the capacity of tumoral cells to escape different types of cellular stresses, such as drug treatments and the host immune response. In this sense, many attempts have been made to find the connection between drug resistance and immune evasion (89), and common pathways controlling both processes have been explored. Within the WNT signaling pathways, many molecules have been described as being the main drivers of the transcriptional activation of the genes involved in the two mechanisms (90–94).

### WNT Signaling in Tumors Drug Resistance

Cancer stem cells develop several resistance mechanisms, which protect the cells from drug damage and make them resistant to chemotherapeutic drugs (11, 12). In parallel with the models explaining tumor heterogeneity, two different proposals account for the existence of cancer drug resistance. According to the clonal evolution model, a population of tumor cells can acquire drug resistance by sequential genetic modifications. After chemotherapy, only the drug-resistant cells within the tumor survive and proliferate, regenerating the tumor bulk made up of the drug-resistant cells progeny. Thus, all tumoral cells became drug resistant. By contrast, the CSC model postulates that after drug exposure, only CSCs (which harbor intrinsic resistance mechanisms) survive. These stem cells then divide and regenerate the tumor mass that will be constituted by stem and differentiated cells, reestablishing the tumoral heterogeneity. The acquisition of drug resistance seems to have features from both models.

Generally, cancer drug resistance involves the participation of a variety of cellular mechanisms, including: drug target mutations; oncogene/onco-suppressor deregulations; activation of pathways blocking the drug action; increased DNA damage repair; overexpression of drug efflux pumps (ABC-transporters); and the induction of cell adhesion-mediated drug resistance, originating due to the crosstalk between tumor and stromal cells. Some of these resistance mechanisms, specifically the over-expression of ABCB1 and CD44 molecules among others, develop because of WNT signaling pathway deregulation in the cancer stem cell subset (Table 1) (104).

**Table 1:** Role of WNT components in drug resistance.

**WNT component**	**Role in drug resistance**	**Type of cancer**	**References**
FZD1 increase	Increased ABCB1 expression	Solid and hematological tumors Human myelogenous leukemia	(95, 96)
FZD1 increase	Increased ABCB1 expression and efflux activity	Neuroblastoma	(97, 98)
FZD7 increase	Increased ABCB1 expression	Hepatocellular carcinoma	(99)
SFRPs methylation	Increased ABCB1 expression	Several types of leukemia Ovarian cancer Cervical cancer Breast cancer	(100)
LGR5 increase	Increased ABCB1 expression	Colorectal cancer	(101)
YAP/TAZ increase	Increased drug resistance	Breast cancer Melanoma	(102, 103)
PYGO2 overexpression	Increased ABCB1 expression	Breast cancer	(104)
APC mutations	Increased CD44v6 expression	Colorectal cancer	(105)
CK1a and GSK3a/b inactivation	Increased drug resistance	Multiple myeloma	(106, 107)
Undefined WNT mediators	Increased CD44 expression	Multiple myeloma	(108)

#### WNT Signaling and Extrusion of Chemotherapeutic Drugs: ABCB1

The superfamily of ABC transporters mediates the efflux or uptake of specific substrates through cellular membranes (plasma membrane, endoplasmic reticulum, Golgi apparatus, peroxisomes, and mitochondria). The number and nature of the substrates is varied and includes physiological and xenobiotic molecules. These transporters are highly conserved and present in prokaryotes and eukaryotes. The ABC transporters are characterized by two transmembrane (TMs) and two nucleotide-binding domains (NBs) or ATP binding cassettes. Human ABC transporters include 48 functional genes classified into seven subfamilies (from A to G) based on different characteristics: similarity in gene structure, order, and sequence homology in the NB and TM domains.

ABCB1 (ATP-binding cassette B1, also known as MDR-1 or P-GP) was the first cloned human ABC transporter. Physiologically, this protein, and other members of the family, transports hydrophobic, and hydrophilic compounds across the placenta, intestine, and other locations and contribute to the blood-brain barrier. Furthermore, this and other transporters actively efflux xenobiotics to protect the cells from cytotoxic agents (11). This includes the capacity to expel a variety of drugs outside the cancer cells, thus inducing chemoresistance in numerous solid tumors and hematological malignancies (13, 109, 110). In that way, ABCB1 contributes to the acquisition of a multidrug resistant (MDR) phenotype since it can bind and extrude a huge repertoire of drugs, thus leading to treatment failure and tumor relapse. Other roles attributed to these proteins include the transport of ABC proteins to intracellular (e.g., cytoplasmic vesicles) or extracellular (e.g., exosomes) compartments to improve drug sequestration (111, 112); tumor cell proliferation, invasion, and deregulation of the pathways involved in apoptosis or complement-mediated cytotoxicity (109, 110, 113–116).

**Figure 3:**
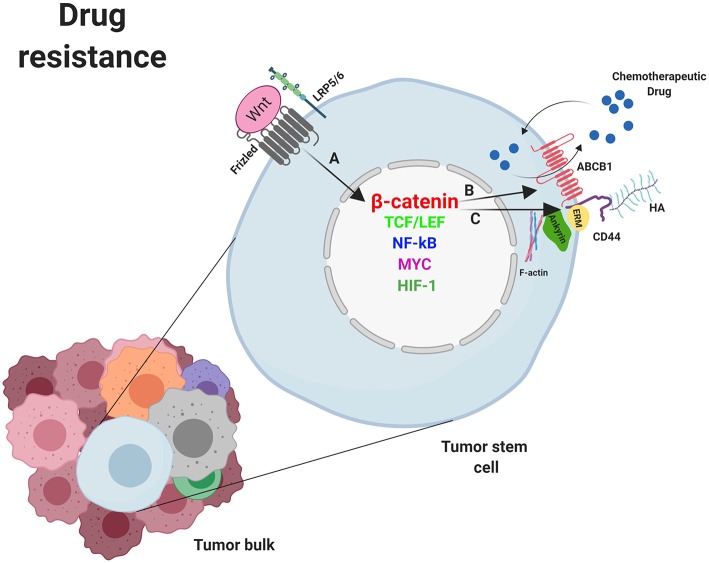
Mechanisms of drug resistance induced by WNT/β-catenin signaling in cancer stem cells. **(A)** Nuclear β-catenin, by means of MYC, TCF/LEF, NF-κB, SNAI1, HIF-1, and other transcription factors, promotes the transcription of genes involved in mechanisms supporting tumor cells survival such as deregulation of molecules involved in drug resistance. **(B)** ABCB-1 and **(C)** CD44. Overexpression of ABCB1 and/or CD44 favor the extrusion of chemotherapeutic drugs out of the cell. ABCB1, ATP-binding cassette B1; ERM, Ezrin, Radixin, Moesin; HA, Hyaluronan; HIF-1, Hypoxia inducible factor 1; LRP5/6, Low-density lipoprotein receptor-related protein 5/6; NF-κB, nuclear factor kappa B; MYC, Myelocytomatosis oncogene cellular homolog; SNAIL, Zinc finger protein SNAI1; TCF/LEF, T-cell factor/lymphoid enhancer factor. Created with BioRender.com.

Stem cells, including CSC, exhibit high expression levels of the two main MDR genes, ABCB1 and ABCG2 (ATP-binding cassette G2) (11). In addition, since the promoter of the ABCB1 gene in humans contains several TCF4/LEF binding motifs, this protein is a target gene of the β-catenin/TCF4 transcriptional regulators. In fact, activation of β-catenin augments ABCB1 expression, which confirms the direct connection between the WNT/β-catenin pathway and chemoresistance. In fact, several studies have demonstrated the existence of this connection in tumor cells which have, in this way, acquired a multidrug resistant phenotype (Figure 3) (117, 118).

##### Frizzled receptors and ABCB1-mediated multidrug resistance

FZD1 was reported to upregulate ABCB1 expression through WNT/β-catenin signaling in solid tumors and hematological malignancies. Both FZD1 and ABCB1 locate in the same region within the 7q21 chromosome. Knockdown of FZD1 induces a significant decrease of ABCB1 protein expression in a human myelogenous leukemia cell line and the recovery of chemosensitivity to antineoplastic drugs by those cells. Either way, the knockdown of ABCB1 using antisense oligonucleotides restored drug sensitivity in the previously resistant cell line (95). Downregulation of FZD1 also resulted in β-catenin degradation and therefore the absence of this protein in the nucleus. Moreover, it has been shown that the knockdown of FZD1 triggers also a decrease in the proliferative capacity of MDR leukemic cells through the WNT/β-catenin cascade. In fact, in cells from patients with acute myeloid leukemia (AML), elevated FZD1 expression at diagnosis was associated with more problems in achieving remission, and a tendency to recurrence. Thus, in patients with relapse, increased FZD1, rather than ABCB1 expression, induced high proliferation and chemoresistance in leukemic cells (96).

WNT/β-catenin signaling deregulation, specifically FZD1 overexpression, has also been observed in neuroblastoma multidrug resistant cell lines. After silencing FZD1 in MDR cells there was inhibition of β-catenin translocation to the nucleus. Consequently, ABCB1 expression and efflux activity were reduced, causing the reversal of the MDR phenotype in those cancer cells (97, 98). Another study explored the role of miR-27a in the modulation of ABCB1-mediated MDR in hepatocellular carcinoma (HCC) using the sensitive cell line BEL-7402 and its resistant counterpart, BEL-7402/5-fluorouracil. The increase in miR-27a levels was associated with enhanced sensitivity of these cells to 5-fluorouracil (5-FU), higher 5-FU-induced apoptosis and downregulation of MDR1/P-glycoprotein expression. That study showed that miR-27a reverses the ABCB1-mediated MDR by downregulation of FZD7 and the inhibition of the WNT/β-catenin pathway (99).

Furthermore, methylation of SFRPs (secreted frizzle-related protein), which inhibits WNT/β-catenin signaling, was reported in several types of leukemia, and associated with resistance to cisplatin in ovarian cancer and with a poor prognosis in cervical and breast cancers. In fact, overexpression of β-catenin was observed in leukemia cells from patients with methylated *SFRP5*, and a demethylation compound recovered SFRP5 protein expression and reduced ABCB1 protein levels. Thus, altered *SFRP5* gene methylation may participate in the development and/or progression of different types of cancers through WNT signaling pathway activation (100).

##### LGR5 and ABCB1-mediated drug resistance

*LGR5* is another target gene of the WNT pathway as well as a marker of colorectal carcinoma CSCs. Several studies have associated higher LGR5 levels with a poor response of colorectal cancer patients to 5-fluoracil-based treatment. In addition, LGR5 positively modulates the ABCB1 expression in CRC cells. That study, as well as others, demonstrated that in cancer cells, LGR5 promotes stem cell properties such as chemoresistance through the positive regulation of the extrusion pump ABCB1 (101).

##### YAP/TAZ and ABCB1-mediated drug resistance

Like other WNT regulators, the activation of YAP/TAZ supports the survival of CSCs treated with conventional chemotherapy, protecting cancer cells against DNA damage. Thus, YAP/TAZ activation protects breast CSCs from paclitaxel, doxorubicin, cisplatin, and radiations and favors resistance to therapies targeting certain molecules in tumor cells with specific oncogenic alterations. Lin et al. reported that YAP confers resistance to inhibitors of RAF and MEK signaling pathways in tumoral cell lines containing activating mutations in *BRAF, KRAS* or *NRAS* genes (102). Conversely, the decrease of YAP enhanced RAF and MEK inhibitor efficacy in mutant cells resistant to monotherapy with those inhibitors. Furthermore, the YAP mechano-transduction pathway is involved in resistance in melanoma and breast cancer (103).

##### Pygopus and ABCB1-mediated drug resistance

PYGO2 is overexpressed in several types of cancer (breast, ovarian, lung, glioma, and esophageal squamous cell carcinoma), and plays a decisive role in the carcinogenesis of these tumors. In fact, PYGO2 was the most upregulated gene in chemo-resistant breast cancer cells. Experimental results indicated that PYGO2 upregulated ABCB1 expression in resistant cells through the WNT/β-catenin cascade. As expected, PYGO2 inhibition restored drug sensitivity in MDR cells by decreasing ABCB1 expression, reducing the breast cancer stem cell subset following chemotherapy. Furthermore, RNA samples from tumors extracted by surgery from 64 paired patients significantly increased PYGO2 and/or ABCB1 expression after chemotherapy, thus underlining a crucial role for the WNT/β-catenin pathway mediated by PYGO2 in the clinical chemoresistance of breast cancer (104).

#### WNT and Activation of Cell Adhesion-Mediated Drug Resistance (CAM-DR): CD44

The constant interaction between the tumor and the surrounding microenvironment influences the cellular fate of CSCs. The protein CD44 is especially important in this communication process since it constitutes a platform for signaling that incorporates cellular microenvironmental information. This molecule integrates signals from growth factors, cytokines and others, and sends them to cytoskeletal proteins associated to the membrane or the nucleus where gene expression is regulated. CD44 upregulates the expression of cell cycle proteins inhibitors, anti-apoptotic proteins, and ABC transporters, and others, thus inducing a form of drug resistance known as cell adhesion-mediated drug resistance (CAM-DR).

The CD44 family is constituted by single-pass, glycosylated class-I transmembrane proteins of 85–90 kDa. The extracellular N-terminal portion of CD44 proteins binds to the glycosaminoglycan hyaluronan (HA), among other ligands, while the C-terminal cytoplasmic tail binds the cytoskeletal linker proteins ezrin, radixin, moesin (ERM), and ankyrin (119), and F-actin (120–122).

The different members of the CD44 family are originated by the alternative splicing of 10 exons which codified for the extracellular domain of the protein. This domain can be completely deleted, generating CD44s or giving rise to various combinations encoding CD44 variant members (CD44v). Although CD44 is found in many normal cell types, it is used as a surface marker for CSCs from several types of tumors (11).

CD44 is a positive regulator of the WNT/β-catenin signaling pathway through the control of LRP5/6 phosphorylation and its location at the plasma membrane (105). Therefore, the ability of CD44 to bind to the cytoskeleton might provide a platform necessary for the interaction between LRP5/6 and kinases such as GSK3β and CK1. Furthermore, the shuttle of LRP5/6-charged vesicles from the Golgi to the membrane might use the F-actin tracks anchored to the plasma membrane through the complex CD44-ERM (123).

Several articles have been published supporting a role for CD44 in CAM-DR. Thus, overexpression of CD44v6 associates with colorectal cancer in advanced stages and is characterized by mutations in the WNT pathway (e.g., APC mutations) (105). Additionally, it was reported that the degree of cell adhesion in MM showed a negative correlation with the sensitivity of these cells to doxorubicin (106). Furthermore, long term exposure of human multiple myeloma cell lines (HMCLs) to lenalidomide increased the level of resistance to this molecule (107) and the expression and activity of β-catenin with enhanced transcription of the WNT target genes cyclin-D1 and myelocytomatosis oncogene cellular homolog (*MYC*). These effects were the consequence of CK1α suppression, and GSK3α/β inactivation, since an increase in the phosphorylation of the inhibitory residues of this protein was observed. In another study in MM, CD44 was identified as the main effector molecule of lenalidomide resistance mediated by the WNT cascade. Overexpression of this protein was observed in lenalidomide-resistant human multiple myeloma cell lines (HMCLs), and in correlation with that, increased adhesion to bone marrow (BM) stromal cells was detected, indicative of CAM-DR. Inhibition of CD44 reduced the adhesion of MM cells and reversed the resistance to lenalidomide (108).

CD44 associates with ABC pumps (124, 125) such as the ABCB1 protein and regulates its gene expression (Figure 3). The process requires the activation of CD44 by HA binding, which promotes PI3K activation and stimulates HA production and ABCB1 expression (126). Alternatively, the binding of HA to CD44 induces the expression and activation of the transcription factor p300 which, together with β-catenin and NFκB-p65, sustains *ABCB1* transcription (127). Engagement of CD44 and activation of specific transcription factors in EMT can also induce apoptosis resistance. Thus, it was demonstrated that the CD44–HA interaction to PKCε induced transport of Nanog to the nucleus, leading to miR-21 synthesis and upregulation of ABCB1 and apoptosis inhibitors (128, 129).

The association between ABCB1/P-GP and CD44 has also been demonstrated by experiments of co-immunoprecipitation and co-localization within the plasma membrane, and further confirmed in a yeast two-hybrid system. In another study, the examination of primary and metastatic tumor samples from osteosarcoma patients showed that a high expression of CD44 was associated with osteosarcoma metastasis and recurrence and CD44 was considered as a solid predictor for chemotherapy response and overall survival in osteosarcoma patients. The results in osteosarcoma cell lines with constitutive knockout of *CD44* gene by CRISPR/Cas9 system verified that CD44 mediates migration, invasion, proliferation and drug resistance to doxorubicin in osteosarcoma cells. In another study, the downregulation of CD44 protein by siRNA in cancer cells from patients with ovarian carcinoma confirmed that the mRNA levels of CD44 and ABCB1 correlate positively. In summary, numerous studies support the hypothesis that CD44 may contribute to tumor drug resistance by regulating ABCB1 expression (130).

### WNT Signaling in Immune Escape by Tumors

The host immune system constitutes a wall against tumor formation through the innate and the adaptive immune response. The primary role of immune effectors such as macrophages, dendritic cells (DCs), and T cells is to discriminate healthy cells from pathogens or tumoral cells through receptors on their cell surface. These molecules integrate all the signals received from microorganisms and/or cells and switch the balance to activation or inhibition of the immune response. Nevertheless, cancer cells can evade detection by the immune cells through the expression of surface molecules that mimic the signals released by healthy cells. Thus, tumoral cells prevent the arrival of immune effectors to the tumor area or, in the event that effector cells infiltrate the tumor, induce their inactivation and death. These molecules expressed by healthy or tumoral cells to keep the immune system under control and their receptors are collectively called checkpoint proteins. These “immune checkpoints” normally function to control excessive immune activation but are also used by tumors to evade the immune system. Because of this, tumors can be classified based on the existence or lack of a T-cell-inflamed tumor microenvironment, and this phenotype correlates with the response to an immune-checkpoint blockade. An inverse correlation has been observed between WNT/β-catenin pathway activation and T-cell infiltration across most human cancers (131, 132). The connection between immune exclusion and the WNT/β-catenin pathway was identified for the first time in metastatic melanoma. When comparing samples categorized as either T cell–inflamed or non-T cell–inflamed it was observed that almost half of the non-T cell–inflamed tumor subset showed increased activation of the WNT/β-catenin signaling pathway. Mechanistic studies using genetically engineered mouse models confirmed that melanomas with increased WNT/β-catenin activation lacked tumor-infiltrating T cells, mimicking the non-inflamed phenotype observed in patients with melanoma. This effect is due to failed recruitment of specific dendritic cells into tumors, leading to impaired recruitment of T cells to the tumor microenvironment (131, 132). Some of the mechanisms involved in the escape of immunity develop after the activation of WNT in tumor cells. Among them, decrease of secretion by tumoral and/or stromal cells of immune cell-attracting chemokines, such as CC-chemokine ligand 4 (CCL4), should be highlighted (61). Other escape mechanisms involve the expression of checkpoint inhibitors such as programmed death ligand 1 (PD-L1) and CD47 which mainly control the activity of tumor-specific T cells and macrophages (61, 133, 134). PD-L1 and CD47 transcription is controlled by MYC, a proto-oncogene identified as a WNT target gene. Thus, mutations in components of the WNT/β-catenin signaling pathway induce aberrant MYC expression and, because of that, increased expression of the immune checkpoint proteins PD-L1 and CD47 and a non-T cell-inflamed tumor phenotype. Conversely, MYC inactivation led to a decrease in the expression of the above proteins, favoring the accumulation of tumor-associated T cells and macrophages (133–135).

#### Impaired DCs Recruitment: CCL4 Inhibition

**Figure 4:**
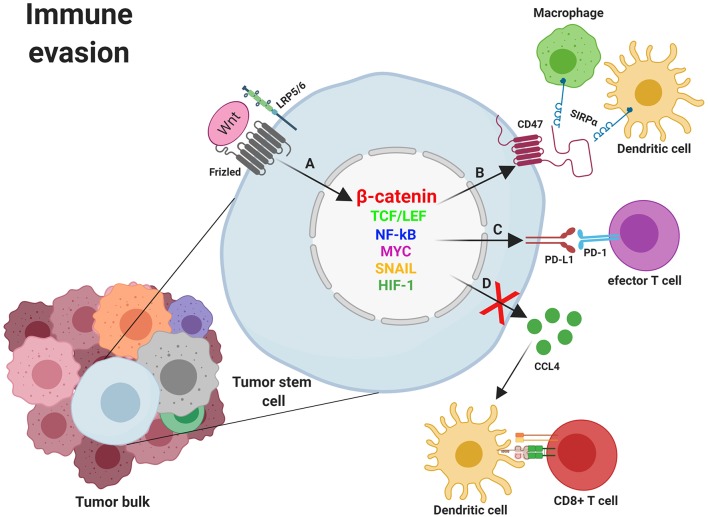
Mechanisms of immune evasion induced by WNT/β-catenin signaling in cancer stem cells. **(A)** Nuclear β-catenin, by means of MYC, TCF/LEF, NF-κB, SNAI1, and other transcription factors, promotes the transcription of genes involved in mechanisms supporting tumor cells survival such as the deregulation of molecules involved in immune evasion (CCL4, CD47, and PD-L1). **(B)** The binding of CD47 to SIRPα might prevent phagocytosis of tumor cells (and/or fragments derived from them) by macrophages and dendritic cells with the subsequent absence of T cell activation. **(C)** Expression of PD-L1 might directly impair effector T cells function within the tumor microenvironment. **(D)** Inhibition of CCL4 secretion by tumor cells avoids Dendritic cells recruitment to the tumor microenvironment and the subsequent T cell priming and activation. CCL4, CC-chemokine ligand 4; LRP5/6, Low-density lipoprotein receptor-related protein 5/6; NF-κB, Nuclear factor kappa B; MYC, Myelocytomatosis oncogene cellular homolog; PD-1, Programmed cell death 1; PD-L1, programmed death ligand 1; SIRPα, Signal regulatory protein Alpha; SNAIL, Zinc finger protein SNAI1; TCF/LEF, T-cell factor/lymphoid enhancer factor. Created with BioRender.com.

Anti-tumoral immune cells such as DCs and T cells need to reach the tumor bulk to perform their anti-tumoral activity. Nevertheless, tumors develop mechanisms to avoid immunity by disrupting chemokine secretion. It has been observed that the secretion of chemokines implicated in effector T-cell recruitment is significantly reduced in several types of tumors lacking dendritic cells and CD8+ T-cell infiltrate. Among the chemokines that act on the recruitment of DCs and whose secretion is reduced in many non-T cell inflamed tumors, CC-chemokine ligand 4 (CCL4/MIP-1beta) should be highlighted (61, 136). As an example, immune exclusion mediated by activation of the WNT–β-catenin pathway was observed in melanoma when expression of CCL4 was inhibited and DCs were no longer recruited into the tumor. Consequently, no T cell priming occurs, and effector T cells were not present at the tumor area (137). Supporting the previous results, a study involving 266 cases of metastatic melanoma found that approximately one third of the melanoma metastatic lesions were non-T cell inflamed and half of them showed activation of WNT–β-catenin signaling in tumor cells. Further support that activation of β-catenin in tumors was related to T cell exclusion was obtained in a genetically engineered mouse model of melanoma which conditionally expressed a dominant stable form of β-catenin. Although all the mice developed melanoma, β-catenin-positive tumors had minimal T cell infiltration and were resistant to therapy based on checkpoint blockade. In β-catenin-positive melanoma cells, secretion of CCL4 and other chemokines was reduced, with insufficient recruitment of specific DCs into the tumor area and defective host priming of antigen-specific T cells (138, 139). Another study in bladder cancer demonstrated the WNT7/β-catenin pathway activation in non-T cell-inflamed tumors (140). Studies in melanoma cells also showed that the CCL4 chemokine and the BATF3 transcription factor were linked to the recruitment of the dendritic cells necessary for T-cell activation (141). Additionally, gene expression of WNT7B was explored in urothelial bladder cancer and an inverse correlation with the presence of CD8 cells was found, further supporting a link between the absence of intratumoral T cells and the activation of WNT signaling. This result was also confirmed by immunohistochemistry. Indeed, detection of CD8 transcripts and BATF3 and CCL4 expression within the tumor area inversely correlated with WNT7B expression (Figure 4) (140).

#### Inhibition of Tumor-Associate Macrophages (TAM) and DCs Phagocytosis Through “Don't Eat Me” Signals: CD47

CD47 (Integrin-Associated Protein, IAP) is a transmembrane glycoprotein, member of the Ig superfamily, with an IgV-like extracellular domain and a short cytoplasmic tail. CD47 expression is ubiquitous in human cells where it is a “marker of self” functioning as a “don't eat me” signal. CD47 has been shown to interact in cis with αvβ3, αIIbβ3, and α2β1 integrins and in trans with thrombospondins (TSPs) and with regulatory molecules belonging to the signal regulatory protein (SIRP) family. SIRPα and SIRPγ are ligands for CD47. SIRPα is highly expressed on myeloid cells (dendritic cells, macrophages, and neutrophils) and smooth muscle cells and discretely expressed by cultured murine and human endothelium. SIRPγ is expressed by T-cells, NK cells, and some B-cells (142).

Interaction of CD47 to SIRP-α promotes the phosphorylation of the immunoreceptor tyrosine-based inhibitory motif (ITIM) on SIRP-α, and the recruitment to the membrane of Src homology region 2 domain-containing phosphatases (SHP-1 and SHP-2), with the subsequent inhibition of myosin-IIA accumulation at the phagocytic area, blocking phagocytosis (143, 144).

CD47 can be expressed on many healthy cells in mice and humans and is highly expressed on a variety of CSCs, including both hematopoietic and solid tumors (145). Expression of CD47 in tumor cells avoid their recognition and elimination by macrophages, dendritic cells, and T cells and induces epithelial-mesenchymal transition (EMT) through modulation of N-cadherin and E-cadherin (146). In fact, CD47 protein expression was significantly high in ovarian cancer and associated with patient stage, chemotherapy resistance, and prognosis. Similar results have been observed in glioma cells, breast cancer cells (147), not small-cells lung carcinoma (NSCLC), PDAC, and others (147–150). The presence of CD47 in AML was also associated with a high self-renewal potential of cancer stem cells and with low patient survival (151, 152). CD47 overexpression also correlates with poor prognosis in head and neck squamous cell carcinoma, melanoma, and osteosarcoma (153–156).

Various transcription factors have been proposed to bind to the promoter of CD47 and explain its upregulated expression in different tumors. Some of them like NF-κB, MYC, SNAI1, ZEB1, HIF-1 and the PKM2–β-catenin–BRG1-TCF4 complex (134, 148, 157, 158) have been implicated in CD47 expression, which strongly suggests that the WNT/β-catenin pathway is involved (Figure 4). Supporting this idea, it has been shown that a constant activation of β-catenin is needed for glioma progression (159), and increased levels of its target genes, such as CD47, have been associated with high-grade GBM (16). Conversely, the inhibition of β-catenin in mutant glioma cells abrogated CD47 expression as well as the interaction between β-catenin and TCF4. The reverse effect was observed in the same cells upon the pharmacological elevation of nuclear β-catenin levels. As expected, the CD47 transcriptional downregulation negatively affected the phagocytosis of cancer cells by microglia (158).

In addition, miRNAs have been described as regulators of stem cells and related with the overexpression of CD47 in cancers. miR-133a acts as a tumor suppressor gene, and is downregulated in many types of tumors (160). miR-133a also regulates the transcription factor TCF7, which is essential in the activation of canonical WNT signaling (161).

In another study, miR-708 induced repression of the WNT/β-catenin signaling pathway in BCSCs, causing inhibition of self-renewal and chemoresistance in these cells. miR-708 was shown to directly bind the CD47 gene regulating the expression of this molecule and the tumor-associated macrophage-mediated phagocytosis (162).

Finally, a role for CD47 in drug resistance has been described. In hepatocellular carcinoma cells (HCC), it was found that clones resistant to sorafenib exhibited increased cancer stem cell characteristics, such as tumorigenicity, self-renewal, and invasiveness. Moreover, an increase in CD47 expression, dependent on nuclear factor kappa B (NF-κB) activation, was found. The knockdown or blocking of CD47 in sorafenib-resistant HCC cells consistently demonstrated an increased sensitization to sorafenib by these cells, suggesting that CD47 signaling might be involved in the sensitization to this drug. Therefore, CD47 may have a role through many as yet unknown pathways in drug resistance (163).

#### Elimination of Activated T Cells Through “Don't Find Me” Signals: PDL-1

PD-L1 is a type I single-pass transmembrane protein, member of the B7 family, organized in an IgV-like domain, an IgC-like domain, and a cytoplasmic tail (163, 164). PD-L1 interacts with the receptor, programmed cell death 1 (PD-1), on activated cytotoxic T cells through the IgV domain. Next, PD-1 forms aggregates with TCR and costimulatory receptor CD28 and recruits the SHP2 phosphatase, leading to its dephosphorylation and inactivation (165, 166). Last, effector T cells become exhausted by the decreased phosphorylation of crucial signaling molecules which regulate activation and proliferation mediated by NFAT (165–167). PD-L1 is expressed in many cell types including macrophages and dendritic cells (164), and tissues such as heart, lung, and placenta (168), and is also overexpressed with immune activation (169). The PD-L1/PD-1 interaction keeps the balance between tolerance and autoimmunity and its deficient or excess functioning can trigger several diseases, including auto-immune diseases such as arthritis and lupus (170). PD-L1 expression has been found to be positive in 5–40% tumor cells such as lung, colon, melanoma, bladder and renal and hepatocellular carcinomas, head and neck cancers, ovarian cancer, and hematologic malignancies (171), inducing elimination of effector cells through interaction of PD-L1 on the surface of cancer cells with PD-1 on the T cells plasma membrane (171–174). It has also been shown that PD-L1 expression is modulated by the WNT pathway (Figure 4). Thus, Triple Negative Breast Cancer Stem Cells (TNBCSCs) exhibit PD-L1 overexpression through the WNT cascade and the upregulation or downregulation of this cascade significantly affects the expression of this molecule (175). In testicular germ tumors β-catenin was described as a marker of poor outcome and a positive correlation with PD-L1 expression was observed, as was a decrease in immune infiltration (176). In melanoma cells, the activation of WNT/β-catenin results in the absence of T cell infiltration in the tumor microenvironment. This effect appears to be a consequence of the action of the negative regulatory pathway PD-L1-PD-1 (141). Finally, an increase in PD-L1 in the population of breast mesenchymal-like cancer cells, but specially in breast CSCs, has been observed during EMT by the EMT/β-catenin/STT3/PD-L1 signaling axis. Specifically, it has been shown that the ER-associated N-glycosyltransferases STT3A and STT3B are required for PD-L1 induction through regulating its glycosylation. This produces PD-L1 stabilization by antagonizing β-TrCP-dependent proteasome degradation of this molecule. In this study, the authors also identified that etoposide suppressed the EMT/β-catenin/STT3/PD-L1 axis through TOP2B degradation-dependent nuclear β-catenin reduction, leading to PD-L1 downregulation of CSCs and non-CSCs, and sensitization of cancer cells to anti-Tim-3 therapy. (177). In summary, several studies support an association between WNT/β-catenin signaling and immune evasion through the regulation of the “don't find me signal” PD-L1 and other molecules involved in immune control.

## New Strategies for Effective Antitumoral Therapy

Most of the anticancer chemotherapies currently being used work by killing highly proliferating cells, which, in many tumors, are mostly non-CSCs, thus decreasing the tumor size. However, the small CSC population present in the tumor bulk is constituted by relatively slow cycling quiescent cells with a higher repair mechanism and are innately resistant to therapy. Furthermore, radiation and chemotherapy may trigger cellular stress response mechanisms enhancing stemness characteristics in non-CSCs and thereby increasing their capacity for adaptation and survival. Thus, conventional chemotherapy can increase the fraction of CSCs within a cancer, making them resistant to treatment, and re-establishing tumor growth in the same or in a distant location from the primary tumor, leading to the formation of metastases which are usually far more resistant to chemotherapy than primary tumors. This has been shown following the treatment of different tumor types, including brain, head and neck, lung, breast, and liver, amongst others (178). Therefore, in contrast to previous therapeutic approaches, new treatments need to consider strategic combinations that could kill CSCs and non-CSCs at once as well as preventing the transition from non-CSCs to CSCs.

Several anti-neoplastic drugs targeting specific molecules are currently in clinical use. One type includes inhibitors of signaling molecules such as inhibitors of tyrosine kinases, Raf, MEK, PI3K, mTOR (mammalian target of rapamycin), and WNT/β-catenin signaling pathways, which are essential for the proliferation of tumoral cells. Another type of therapy is based on obtaining and using monoclonal antibodies (mAbs) to surface proteins highly expressed in cancer cells, such as CD20 in lymphoma (rituximab), HER2 in breast cancer (trastuzumab), and epidermal growth factor receptor (EGFR) in colon cancer (cetuximab). Another type of therapy includes compounds and/or mAbs targeting molecules with a high expression in CSCs and with an essential role in drug resistance and/or tumor immune escape. Inhibitors and antibodies (Abs) specific for ABCB1, CD44, CD47, and/or PD-L1 belong to this group. The great heterogeneity of tumor cellular composition and the complex mechanisms involved in the tumorgenicity in CSCs and non-CSCs subsets requires the use of many different therapeutic approaches and targets, based on the characteristics of the tumor and the patient. Many of these approaches developed to eliminate tumor bulk are used in combination and are currently under clinical evaluation.

### Targeting Molecules Involved in WNT Pathway

**Figure 5:**
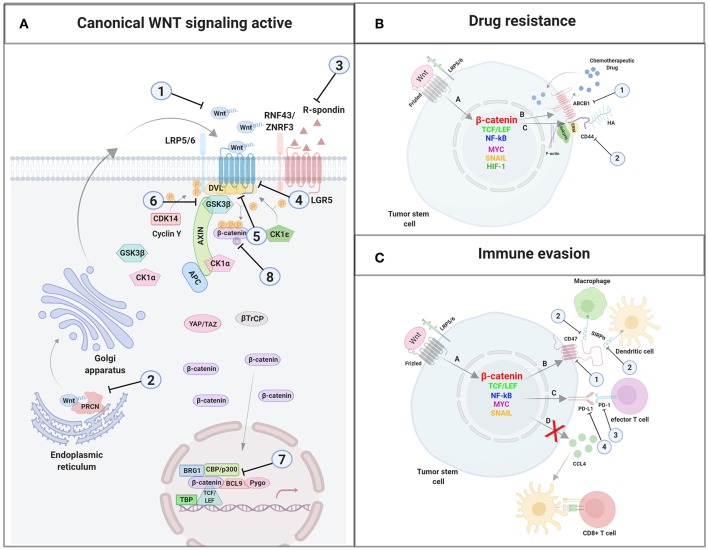
Inhibitors used for antitumoral therapy. **(A)** Agents targeting molecules of the WNT canonical pathway: (1) Targeting WNT ligands or agonists; (2) Porcupine specific inhibitors; (3) Blocking antibody targeting RSPO3; (4) Frizzled blocking agents; (5) Agents targeting LGR5; (6) GSK3β inhibitors; (7) Transcription complex inhibitors; (8) Agents targeting β-catenin expression. **(B)** Agents targeting molecules involved in drug resistance: (1) Inhibitors of the extrusion pump ABCB1; (2) Blocking antibodies targeting CD44. **(C)** Agents targeting molecules involved in immune evasion: (1) CD47 blocking agents; (2) Agents targeting SIRPα; (3) Agents targeting PD-1; (4) Agents targeting PD-L1. Created with BioRender.com.

Targeting the WNT signaling pathway in CSCs is an interesting and promising approach for anti-tumoral therapy. Although this signaling pathway is expressed in normal cells, deregulation is found in CSCs. Compounds with WNT inhibitory properties can target the CSCs but also normal cells, inducing adverse effects. In this regard, therapeutic molecules should be modified or combined with other therapies to improve their specificity and efficiency. Thus, current chemotherapeutic strategies focus on targeting WNT signaling in specific tumor subclasses or with specific mutational characteristics. Ideally, this tumor-targeted therapy should have at least, three main effects: first, recruitment of dendritic cells, macrophages, and effector T cells in the tumor area; second, reversal of resistance to tumor drugs; and third, inhibition of tumor evasion. Since canonical WNT signaling promotes not only T cell lymphopoiesis but also regulation of peripheral immune cells activation and differentiation (61, 72), ongoing therapy should also have a minimal incidence on immune cells, specifically those infiltrated in the tumor area. Otherwise, mechanisms of anti-tumoral immunity will not function properly and the effectiveness of the immune cells fighting the tumor will be seriously compromised. Finally, given the crucial role of WNT signaling in the maintenance of stem cells and the regeneration of tissues and organs in homeostasis, unwanted side effects should be carefully evaluated. In fact, although diverse types of WNT/β-catenin pathway inhibitors are under development as anti-neoplastic therapies in many hematologic and solid malignancies, none have been approved for clinical use (Figure 5A) (36).

#### Targeting WNT Ligands

Monoclonal antibodies, recombinant proteins, and other inhibitors that neutralize WNT ligands or agonists of these molecules are under clinical investigation and have demonstrated WNT inhibition and tumor reduction in melanoma, sarcoma, colorectal cancers, non-small cell lung carcinoma, and mesothelioma. For example, a hexapeptide has been synthesized which can imitate the characteristics of the WNT5a ligand and inhibit tumor cell migration *in vitro*. That compound is being tested in a phase 1 study of patients with breast, colon, and prostate metastatic cancers (36, 37). Moreover, a WNT inhibitor which binds and sequesters WNT, blocking the signaling cascade, is in Phase 1b clinical trials (clinicaltrials.gov). Additionally, specific inhibitors have been found that neutralize Porcupine, preventing acylation, and WNT secretion (179). These inhibitors have been tested in phase I trials in melanoma, breast cancer, and pancreatic cancer, metastatic colorectal and head and neck cancers. Promising therapeutic results have also been obtained in pre-clinical models of CRC with another treatment, based on the use of a blocking antibody targeting RSPO3 which produces a loss of stemness and differentiation.

#### Targeting Frizzled Receptors and Co-receptors

Several trials testing Abs specific for receptors and co-receptors of the WNT signaling pathway are currently in process. For example, an anti-Fz10 radiolabeled mAb is being evaluated for the treatment of synovial sarcoma in a phase I clinical trial. Also, a monoclonal antibody specific to five human FZD receptors inhibits the growth of human tumor xenografts in mouse models. This antibody is currently included in phase I clinical trials used as monotherapy or in combination with taxanes in breast and pancreatic cancer and non-small cell lung cancer. It is also being used in combination with conventional chemotherapy in phase 1b clinical trials with breast, liver, ovarian, pancreatic, and lung cancer patients (180). Additionally, the use of Abs conjugated to cytotoxic drugs targeting LGR5 has demonstrated therapeutic effectiveness in xenografts and in genetic mouse models of CRC.

#### Disheveled Inhibitors

A non-steroidal anti-inflammatory drug (NSAID) which targets DVL is currently undergoing testing in phase II trials, although multiple non-NSAID inhibitors of DVL have also been developed. Specifically, these agents block the binding between FZD and DVL at the membrane, inhibiting DVL activation. As a consequence of this, the destruction complex is stabilized, thus promoting β-catenin degradation (12, 151, 181).

#### GSK3β Inhibitors

The effect of an inductor of GSK3β degradation was tested in combination with chemotherapy on cholangiocarcinoma (CCA) cells and was shown to enhance the effect of classical chemotherapy. This agent downregulated ABCB1 expression in a GSK3β-dependent manner (182). In addition, lawsone derivatives are compounds which induce collateral sensitivity in MDR ABCB1-overexpressing cells. These compounds decrease β-catenin activity in a reporter cell line and downregulate c-MYC, ABCB1/P-GP, and FZD7 protein expression. Furthermore, WNT/β-catenin signaling was selectively inhibited in resistant cell lines (108).

#### TCF/CBP/β-Catenin Transcription Complex Inhibitors

Another inhibitor of WNT/β-catenin signaling that has been synthesized specifically binds to the N-terminus of CBP and not p300, blocking only the interaction between CBP and β-catenin. The compound leads to differentiation and loss of self-renewal capacity in pre-B ALL cells. Also, this drug downregulated survivin, an inhibitor-of-apoptosis protein, (IAP) in primary ALL and, in combination with conventional therapy and independently of the mutational status of CBP and chromosomal aberration, eradicates drug-resistant primary leukemia *in vitro* and prolongs the survival of NOD/SCID mice inoculated with primary ALL (183–185).

Additionally, the use of RNA interference technology to knock down the expression of β-catenin results in the reduction of many CSC properties, such as proliferation, migration, drug resistance, and expression of transcription factors such as OCT-4, amongst others (77).

Also, CRISPR/Cas9 genome editing technology has been used to delete β-catenin. Thus, results have shown reduced tumorigenesis in mixed lineage leukemia and regression of epidermal tumors by depleting CSCs.

Another experimental strategy involves the activation of quiescent CSCs subsets which increases their sensitivity to chemotherapeutic drugs. Quiescence has been associated with the activity of the ubiquitin ligase FBXW7, which downregulates MYC levels. Thus, in experimental models of chronic myeloid leukemia (CML), genetic ablation of *FBXW7* in quiescent leukemic stem cells reprograms them to re-enter the cell cycle and the cells become susceptible to imatinib (186).

### Targeting Molecules Involved in Drug Resistance: ABCB1 and CD44

#### Targeting ABCB1

Several attempts have been made to efficiently counteract the action of the extrusion pump ABCB1/P-GP and although many pump extrusion inhibitors have been assayed in clinical trials, none of them have been approved for use in patients. Currently, the third and fourth generations of ABCB1/P-GP inhibitors are being designed and/or tested. Third generation inhibitors use nanomolar concentrations to increase their effectiveness at reversing MDR compared to first- and second-generation compounds. Thus, elacridar and zosuquidar significantly inhibited ABCB1 with low toxicity in phase I clinical trials. More specifically, zosuquidar, an oral ABCB1 inhibitor, has been used in the treatment of acute myeloid leukemia, and improves the uptake of daunorubicin, idarubicin, and mitoxantrone. Another inhibitor, tariquidar, has elevated ABCB1 affinity and reduces the ATPase activity of the extrusion pump. Nevertheless, phase III assays of tariquidar with carboplatin/paclitaxel or with vinorelbine were closed due to toxicity issues. Tetrandrine, an alkaloid isolated from *Stephania tetrandra*, modulates the activity of ABCB1 and has also been used with doxorubicin in phase I clinical trials (www.clinicaltrials.gov) in the treatment of multidrug resistant cancers (187).

More recently, researchers have been working on the fourth generation of ABCB1 inhibitors, which are natural compounds derived from plant extracts, marine organisms, fungi and other sources, and exhibit modulatory properties on ABCB1, less cytotoxicity, and better oral bioavailability. The catalog of these compounds includes alkaloids, coumarins, flavonoids, and terpenoids, many of which are ABCB1 inhibitors. Thus, trabectedin, cytarabine, and Halaven have been approved for use with patients due to their powerful ABC drug transport reversal activity (188). Other phytochemicals such as curcumin and quercetin prevent ABCB1 function reversing MDR in human cancer cell lines (189, 190). Another example is piperine, an alkaloid and major component of black pepper (*Piper nigrum*) and long pepper (*Piper longum*). This compound exhibits P-GP inhibitory activity, and also antitumoral, antioxidant, antimicrobial, and hepatoprotective functions (191). Other flavonoids such as luteolin and casticin have been shown to be active on glioma stem-like cells (192, 193). 8-Bromo-7-methoxychrysin (BrMC) induces apoptotic cell death on hepatocellular carcinoma (Hep-G2 cell line) by generating reactive oxygen species (ROS) (194). Additionally, LY294002 is a compound with a PI3K inhibitory activity that induces apoptosis in osteosarcoma CSCs by blocking the cell cycle. This compound also inhibits ABCG2/BCRP, ABCB1/MDR1/P-GP, and ABCC1/MRP1, three transporters with a high expression in stem cells. In addition, salinomycin, an antibiotic isolated from *Streptomyces albus*, interferes with ABC transporters and CSC pathways in many solid tumors and hematological cancers (187, 195).

Thus, novel compounds are being considered as new extrusion pumps inhibitors. Nevertheless, many adverse effects must be considered. Inhibition of efflux pumps can cause adverse effects in normal stem cells, since they exhibit high expression of several extrusion pumps which they need for their physiological function. In addition, ABCB1, and other pumps, play an essential role in keeping the integrity of the blood brain barrier, and interfering with their normal function could have negative consequences for the patient's health (Figure 5B) (187, 196, 197).

#### Targeting CD44

Another promising approach to blocking essential CSC signaling pathways is the use of therapy based on Abs. Thus, several anti-CD44 monoclonal Abs have been obtained with very promising results, since some of them selectively eliminate the self-renewal properties of CSCs in several malignancies and cancer cell lines. Specifically, the antibody H90 can discern between the stem cells from conventional hematopoietic progenitor cells and from AML cells. Also, the antibody P245 inhibits estrogen and progesterone receptor and HER2 in TNBCC and H4C4 blocks tumor sphere formation in human pancreatic carcinoma cells and inhibits tumorigenesis in a murine xenograft model. Notably, another recombinant and humanized anti-CD44 monoclonal antibody (RO5429083/RG7356) blocks tumorigenesis in head-and-neck carcinoma cells in mice by the cytolytic action of natural killer (NK) cells. Moreover, this antibody can eliminate triple-negative MDA-MB-231 breast cancer and CLL cells (Figure 5B) (198).

### Targeting Molecules Involved in Immune Evasion: CD47 and PD-L1

#### Targeting CD47

The interaction CD47-SIRPα has been intensively investigated as potential cancer therapy (199–201). Several humanized monoclonal Abs have been obtained with encouraging therapeutic results. For example, Hu5F9-G4, the first humanized mAb to human CD47 increases the phagocytosis of tumor cells by macrophages *in vitro* and eliminates tumors in xenograft mouse models. The humanized mAb to human CD47, Hu5F9-G4, and CC-90002, are being tested in phase I or I/II clinical trials for solid tumors and hematological malignancies (202). Treatment with another anti-CD47 antibody, B6H12.2, has been shown to improve CD8+ T-cell cytotoxicity and macrophages phagocytosis and decrease tumorigenesis in AML CSCs in animals. Additionally, promising results have been obtained in many types of cancer cells. Therapy based on the use of B6H12 also decreased tumor formation in a leiomyosarcoma mouse model and the reduction of CSCs in pediatric brain carcinomas (151).

Moreover, another therapeutic approach is the design and synthesis of engineered recombinant SIRPα proteins with improved affinity for the CD47 molecule blocking endogenous CD47-SIRPα interaction. Specifically, TTI-621 is formed by the Ig-V-like domain of human SIRPα linked to the Fc region of human IgG1 (203). Similarly, the recombinant protein ALX148 is constituted by a variant of the Ig-V-like domain of human SIRPα anchored to an inactive Fc domain (203, 204). Both treatments are being used in hematological malignancies or solid tumors; as single medication or with Abs specific for tumoral antigens, with radiotherapy or with immune checkpoint inhibitors. CD47 blockade promotes antibody-dependent cellular phagocytosis (ADCP) of tumor cells by macrophages and tumor killing by cytotoxic T lymphocytes. In contrast, since many cell types express CD47, therapy based on targeting that molecule could induce adverse effects and, in fact, treatment with anti-CD47 Abs induced the appearance of anemia in monkeys (Figure 5C) (204).

#### Targeting SIRPα

Another potentially successful strategy for cancer therapy is the combination of a molecule that blocks SIRPα with Abs specific for tumoral antigens. In fact, the combined use of anti-SIRPα murine Abs and rituximab, eliminated human Raji cells grafted into non-obese diabetic (NOD)/SCID mice (205). Moreover, blocking CD47-SIRPα interaction with an antibody specific for human SIRPα enhanced killing *in vitro* by macrophages of HER2+ breast cancer cells previously opsonized with the anti-HER2 monoclonal antibody, trastuzumab (206).

The therapeutic success of anti-SIRPα blocking Abs in tumors SIRPα-positives, might be based on the activation of the ADCP mechanism against tumor cells by macrophages together with the elimination of the phagocytosis blockade exerted by the CD47-SIRPα interaction. In fact, the use of a murine Ab anti-SIRPα reduced tumorigenesis in mice inoculated with cells from renal cell carcinoma or melanoma (205). Cytotoxic T cells and NK cells could also participate in the antitumor mechanisms triggered by the anti-SIRPα Abs (205). Such Abs could favor activation of cytotoxic T lymphocytes by the macrophages and DCs infiltrated in the tumor area and induce antibody-dependent cellular cytotoxicity (ADCC) by NK cells toward tumor cells (206–208). It is of great importance that this type of treatment has thus far not induced adverse effects such as anemia or neurotoxicity in mice (206). In addition, recombinant CD47 proteins blocking the interaction between CD47 and SIRPα may also contribute to the elimination of tumor cells. Indeed, a variant of CD47 with improved affinity for SIRPα had a synergistic effect when used in combination with tumor-specific monoclonal Abs to ameliorate phagocytosis of tumor cells *in vitro* (209), although its antitumoral effects *in vivo* have not been evaluated (Figure 5C).

#### Targeting PD-L1

Therapies based on anti-PD-1 and anti-PD-L1 Abs have been designed to augment the cytotoxic T-cell attack to tumor cells. However, these treatments sometimes fail because of the emergence of resistance in patients. Furthermore, these Abs sometimes do not target all the CSCs present in the tumor bulk or induce insufficient signaling responses. To solve these problems, researchers have obtained multi-specific Abs with the capacity to bind more than one membrane receptor. These chimeric Abs are very efficient at blocking ligand binding and inhibiting downstream signaling. Several inhibitors of PD-L1 have been obtained such as atezolizumab, durvalumab, avelumab, and inhibitors of PD-1 include nivolumab and pembrolizumab (168). Treatment of patients with PD-1 and PD-L1 inhibitors, alone or in combination with standard chemotherapy, has demonstrated preliminary positive results in advanced TNBC (151, 175).

Finally, CRISPR/Cas9 is another method to cleave DNA, allowing edition in any cell. A clinical trial based on this technology was initiated to combat non-small-cell lung cancer by deleting the PD-1 receptor (Figure 5C) (151).

#### Combination of Treatments to Target Several Immune Checkpoint Inhibitors

Combinations of therapies based on targeting immune molecules with standard chemotherapeutic drugs and novel therapeutic agents could improve the efficacy of immune checkpoint inhibition in unresponsive patients. The strategy is based on the joint use of Abs and/or recombinant proteins targeting checkpoint inhibitors, chemotherapeutic drugs, cancer vaccines, and immune-stimulatory molecules. These combination therapies are currently being tested in pre-clinical models and patients. The joint blockade of the CD47-SIRPα and PD-1-PD-L1 interactions might have a synergistic effect in the elimination of tumor cells. Indeed, an anti-CD47 nanobody that inhibits the CD47-SIRPα interaction synergized with a PD-L1 antagonist significantly reducing the growth of tumors in mice previously injected with melanoma cells (210, 211). The efficacy of therapy based on the combined use of anti-PD-1 and anti-CTLA-4 antibodies was enhanced by using anti-CD47 blocking Abs in a murine model of esophageal squamous cell cancer (210). Moreover, combined therapy with SIRPα and PD-1 blocking agents had a synergistic antitumoral effect in a murine model of colon cancer (205). The combination of agents with capacity to inhibit the CD47-SIRPα interaction with Abs that block the PD-1-PD-L1 binding is a promising therapeutic approach for the treatment of a broad range of cancers (144, 210, 211).

## Conclusions

WNT/β-catenin signaling is a highly conserved pathway involved in multiple essential homeostatic functions. Nevertheless, deregulation of this pathway is involved in cancer progression, drug resistance, and immunity escape. Thus, at the same time the WNT pathway is activated in CSCs, a complex transcriptional program is initiated, resulting in the expression of genes such as *ABCB1* and *CD44*, which are involved in drug resistance, and *PD-L1* and *CD47*, which are well-known in immunity. This increased WNT activation in tumor cells is responsible for the start of different survival programs which have made tumor cells highly resistant to the anti-tumoral response and/or the drug treatment. For a long time, inhibitors and/or blocking Abs have been used to counteract the action of ABCB1 as an extrusion pump or to block PD-L1 and/or CD47, and combined therapy has been relatively successful. Additionally, many components of the WNT pathway have been the focus of the scientific community as promising therapeutic targets to eliminate tumor cells. Nevertheless, although the combination of several therapeutic approaches has been promising, many challenges remain to be solved in order to design an effective anti-tumoral treatment which targets tumoral cells, including the small population of CSCs that constitutes a critical subset of the tumor bulk.

## Author Contributions

EM-O wrote and revised the manuscript and supervised the figures and references. AS-F assisted in the elaboration of the figures and references list. IO-P organized the references list. All authors assisted in the conception of this review, acquisition of relevant literature, and gave their approval of the last version to be published.

### Conflict of Interest

The authors declare that the research was conducted in the absence of any commercial or financial relationships that could be construed as a potential conflict of interest.
